# Evaluating radiological response in pancreatic neuroendocrine tumours treated with sunitinib: comparison of Choi versus RECIST criteria (CRIPNET_ GETNE1504 study)

**DOI:** 10.1038/s41416-019-0558-7

**Published:** 2019-09-03

**Authors:** Mª Pilar Solis-Hernandez, Ana Fernandez del Valle, Alberto Carmona-Bayonas, Rocio Garcia-Carbonero, Ana Custodio, Marta Benavent, Teresa Alonso Gordoa, Bárbara Nuñez-Valdovino, Manuel Sanchez Canovas, Ignacio Matos, Vicente Alonso, Carlos Lopez, Antonio Viudez, Marta Izquierdo, David Calvo-Temprano, Enrique Grande, Jaume Capdevila, Paula Jimenez-Fonseca

**Affiliations:** 1 Medical Oncology Department, Hospital Universitario Central de Asturias, Oviedo, Spain; 2 Radiology Department, Hospital Universitario Central de Asturias, Oviedo, Spain; 3 Hematology & Medical Oncology Department, Hospital Universitario Morales Meseguer, UMU, IMIB, Murcia, Spain; 4 Medical Oncology Department, Hospital Universitario 12 de Octubre, imas12, CNIO, UCM, CIBERONC, Madrid, Spain; 5 Medical Oncology Department, Hospital Universitario La Paz, CIBERONC CB16/12/00398, Madrid, Spain; 6 Medical Oncology Department, Hospital Universitario Virgen del Rocío, Sevilla, Spain; 7 Medical Oncology Department, Hospital Universitario Ramón y Cajal, Madrid, Spain; 8 Medical Oncology Department, Hospital Clínico de la Universidad de Chile, Santiago de Chile, Chile; 9 Hematology & Medical Oncology Department, Hospital Universitario Morales Meseguer, Murcia, Spain; 10 Medical Oncology Department, Hospital Universitario Vall d’Hebron, Vall d’Hebron Institute of Oncology (VHIO), Universitat Autònoma de Barcelona, CIBERONC, Barcelona, Spain; 11 Medical Oncology Department, Hospital Universitario Miguel Servet, Zaragoza, Spain; 12 Medical Oncology Department, Hospital Universitario Marqués de Valdecilla, Santander, Spain; 13 Medical Oncology Department, Complejo Hospitalario de Navarra, Pamplona, Spain; 14 Medical Oncology Department, MD Anderson Cancer Center, Madrid, Spain

**Keywords:** Cancer imaging, Neuroendocrine cancer

## Abstract

**Background:**

The purpose of our study was to analyse the usefulness of Choi criteria versus RECIST in patients with pancreatic neuroendocrine tumours (PanNETs) treated with sunitinib.

**Method:**

A multicentre, prospective study was conducted in 10 Spanish centres. Computed tomographies, at least every 6 months, were centrally evaluated until tumour progression.

**Results:**

One hundred and seven patients were included. Median progression-free survival (PFS) by RECIST and Choi were 11.42 (95% confidence interval [CI], 9.7–15.9) and 15.8 months (95% CI, 13.9–25.7). PFS by Choi (Kendall’s τ = 0.72) exhibited greater correlation with overall survival (OS) than PFS by RECIST (Kendall’s τ = 0.43). RECIST incorrectly estimated prognosis in 49.6%. Partial response rate increased from 12.8% to 47.4% with Choi criteria. Twenty-four percent of patients with progressive disease according to Choi had stable disease as per RECIST, overestimating treatment effect. Choi criteria predicted PFS/OS. Changes in attenuation occurred early and accounted for 21% of the variations in tumour volume. Attenuation and tumour growth rate (TGR) were associated with improved survival.

**Conclusion:**

Choi criteria were able to capture sunitinib’s activity in a clinically significant manner better than RECIST; their implementation in standard clinical practice shall be strongly considered in PanNET patients treated with this drug.

## Background

Pancreatic neuroendocrine tumours (PanNETs) are a heterogeneous, typically slow-growing group of neoplasms.^1^ One of their biological hallmarks is their rich microvascular density that originates in the dense vascular network of the pancreatic islet. This structure is sustained by a host of proangiogenic molecules, such as vascular endothelial growth factor receptors (VEGFR), platelet-derived growth factor receptor (PDGFR) and others.^2,3^ As a result, PanNETs appear as hypervascular masses on computed tomographies (CT), with avid contrast enhancement in the arterial phase.^4^ The intensity of radiological enhancement correlates with the microvascular density.^5^

In the phase III randomised trial SUN1111 conducted in advanced, progressive, grade 1/2 PanNET, the use of the multikinase inhibitor (MKI) sunitinib, which targets VEGFR 1, 2, and 3, as well as PDGFR, improved progression-free survival (PFS).^6,7^ Other antiangiogenic drugs that inhibit the VEGF/VEGFR pathway, such as lenvatinib, pazopanib, cabozantinib, surufatinib or famitinib, are being actively evaluated in PanNETs.^8^ These molecules foster the appearance of altered, meandering tumour vessels that are susceptible to leaks associated with areas of necrosis within the tumour.^9^ The tomographic correlate includes cystic degeneration or intratumoural hypodensity, common radiological findings that are considered evidence of in vivo activity of the antiangiogenic agent.^10^ In contrast, clinical trials with these drugs in PanNETs usually reveal low rates of tumour shrinkage.^6,11^

Response Evaluation Criteria In Solid Tumors (RECIST) were developed to quantify efficacy of cytotoxic chemotherapy according to the degree of tumour regression.^12^ Despite their wide acceptance and reproducibility, their limitations are becoming better known when evaluating the antiproliferative and antiangiogenic effects of targeted molecular agents,^13,14^ especially in slow-growing tumours such as PanNETs.^15–17^ This translates as an underestimation of treatment effect in clinical trials and the inappropriate discontinuation of effective therapies in the case of tumours that maintain hypo-attenuation as a correlate for effective biological activity, despite a discrete increase in diameters.^10,16,18^

In response to these challenges, Choi et al. were the first to propose that the combined variation in tumour density and size, with lower thresholds than the ones used by RECIST, was an appropriate method by which to monitor response to targeted therapies.^19–22^ Comparative data suggest improved performance of Choi versus RECIST criteria in a number of solid tumours including neuroendocrine tumours;^10,18,16^ however, further evaluations are needed to validate this promising preliminary results prior to recommending their widespread use for PanNETs in clinical practice.

Against this backdrop, the CRIPNET_GETNE1504-study (NCT02841865) seeks to compare the usefulness of Choi versus RECIST criteria in well-differentiated, advanced PanNETs and to discern which criterion best captures sunitinib’s activity in this population.

## Methods

### Study design and patients

CRIPNET_GETNE1504 is a multicentre study classified by the Spanish Agency of Medicines and Medical Devices (AEMPS, for its acronym in Spanish) as a post-authorisation trial of prospective follow-up. It was conducted at 10 sites of the Spanish Group of Neuroendocrine and Endocrine Tumors (GETNE, for its acronym in Spanish) and recruited consecutive patients with advanced, well-differentiated PanNETs treated with sunitinib between 2012–2017. The study did not include any kind of intervention. The decision to modify treatment was therefore based on the investigator’s criterion, according to local standard radiological evaluation as per RECIST. The study was approved by the Ethics Review Boards of the participating centres and by the healthcare authorities of each geographical region. All subjects gave their written informed consent to participate in the study.

Eligibility criteria included: histological confirmation of PanNET, Ki-67 index ≤ 20%, availability of serial image studies, with arterial and portal phase, within the 4 weeks prior to initiating treatment with sunitinib and at least every 6 months until progression or the end of the study. Individuals were excluded if they received other concurrent antiproliferative treatments, with the exception of somatostatin analogues prescribed for their anti-secretory effect; those with short exposure to sunitinib (fewer than two cycles) due to causes other than demise or clinical decline, or with previous use of other antiangiogenic agents. Cases with suboptimal or non-standard CT assessment (e.g. insufficient enhancement with contrast, insufficient body coverage), or the absence of measurable disease were likewise excluded.

Sunitinib was started at the standard dose, 37.5 mg/day, and the criteria for dose reductions and delays were done as per the summary of product characteristics and the clinical judgment of the attending medical oncologist, expert in the management of PanNETs.

### Objectives

The main objective of the study was to assess the association of RECIST v1.1 and Choi criteria on survival endpoints. Other aims were to analyse whether PFS as per Choi is a better surrogate endpoint for overall survival (OS) than PFS according to RECIST, and to appraise the correlation with prognosis of attenuation variation rate (AVR), tumour growth rate (TGR) and diameter variation rate (DVR).

### Measures (acquisition, imaging analysis and criteria of evaluation)

Patients underwent contrast-enhanced CT imaging of the chest, abdomen and pelvis. The equipment used in these studies was CT multidetector (64– or 128–detector row scanners). All studies had to be performed with intravenous contrast according to each centre’s standard protocol, including arterial and portal venous phase imaging deemed optimal for accurate evaluation, with a minimum of 5-mm reconstruction intervals. Images were independently read by two radiologists, specialised in NETs, who were blind to local results and to clinical data, and reached a consensus regarding their evaluation of response. PFS was defined as the time in months from initiation of treatment with sunitinib until progression or death, censoring event-free subjects.

Choi and RECIST v1.1 criteria were analysed as per the usual descriptions.^12,23^ The exact definitions followed to interpret the images are displayed in Supplementary Table 1. In keeping with RECIST v1.1, up to five target lesions were selected (maximum of two per organ involved) calculating the sum of target lesions in conformity with pre-established criteria.^12^ The Hounsfield unit (HU) value was obtained using the mean attenuation of the pixels of the region of interest within the contour of the target lesions in arterial phase. The result was averaged to obtain a mean measure of CT attenuation. Changes in size or attenuation were calculated in absolute or relative terms against baseline values and the previous CT. The AVR and DVR were calculated by dividing the relative changes in density and size by the time since commencement of sunitinib until evaluation of response. The TGR was calculated according to the definition by Ferté.^24^ Tumour size was defined as the baseline sum of target lesion diameters (D0), while Dt is the same measure after time (t) has elapsed. Thus, tumour growth (TG) was calculated using the formula: TG = 3 Log(Dt/D0)/t. To express this magnitude in clinically relevant terms, TG was expressed as a percentage of variation over 1 month, by means of the transformation: TGR = 100 (exp(TG) −1).

### Statistics

Cohen’s kappa was used as a measure of concordance between Choi/RECIST criteria. Correlation between PFS & OS was quantified by means of Kendall’s τ associated with Clayton’s copula models for bivariate survival data.^25^ Wilcoxon signed rank test was used for paired data to compare variations in size/attenuation between consecutive radiological studies. A landmark estimation was made for PFS at 24 months, after 6 months of follow-up. The landmark method estimates the likelihood of survival after a set period of time and is suitable for time-dependent variables, such as tumour response.^26,27^ In this case, the landmark coincides approximately with the date of the evaluations of response conducted in the patients. The prognostic effect was appraised by Cox proportional hazards regression, factoring in the time-dependent nature of the evaluation of response. The Mantel–Byar test was used to compare survival endpoints as per Choi or RECIST. This method is a modification of the log-rank test that avoids bias in survival analysis by tumour response.^27^ The analyses were executed with the RStudio statistical software (RStudio, Inc., Boston, MA, USA), including the landest, survival and rgl packages.^28–30^

## Results

### Patients

One hundred and seven patients were recruited, 22 of whom were excluded as they did not meet the quality requirements per protocol for imaging studies, including the adequate predefined frequency of procedures. The baseline characteristics of the 85 individuals included are presented in Table 1. Participants had a median age of 59 years (range, 21–84) and 58% were male. PanNETs had a median Ki67% of 7 (range, 1–20) and in 62%, metastases were limited to a single organ. Sixty-five percent received sunitinib after a somatostatin analogue. Sunitinib was maintained for a median of 52 weeks (range, 7–301), with dose reductions reported in 36% of treated patients.

**Table 1:** Patients’ sociodemographic variables and baseline characteristics

	*N* = 85, %
Sex, male	49 (58%)
Age, median (range)	59 (21–84)
ECOG PS 0/1/2	35/49/1
Functioning tumour	21 (25%)
Sites of metastases	
Liver	80 (94%)
Peritoneum	4 (5%)
Lymph nodes	26 (31%)
Lung	3 (4%)
Other	10 (12%)
Number of involved organs, median (range)	1 (0–4)
Ki67, median (range)	7 (1–20)
Prior SSA	55 (65%)
Previous lines excluding SSA	
0	46 (54%)
1	20 (23%)
2	17 (20%)
3	2 (2%)
Sunitinib as first-line therapy	22 (26%)
Primary tumour surgery	33 (39%)
Locoregional therapies	4 (5%)

### Evaluation of PFS by RECIST versus Choi

**Fig. 1:**
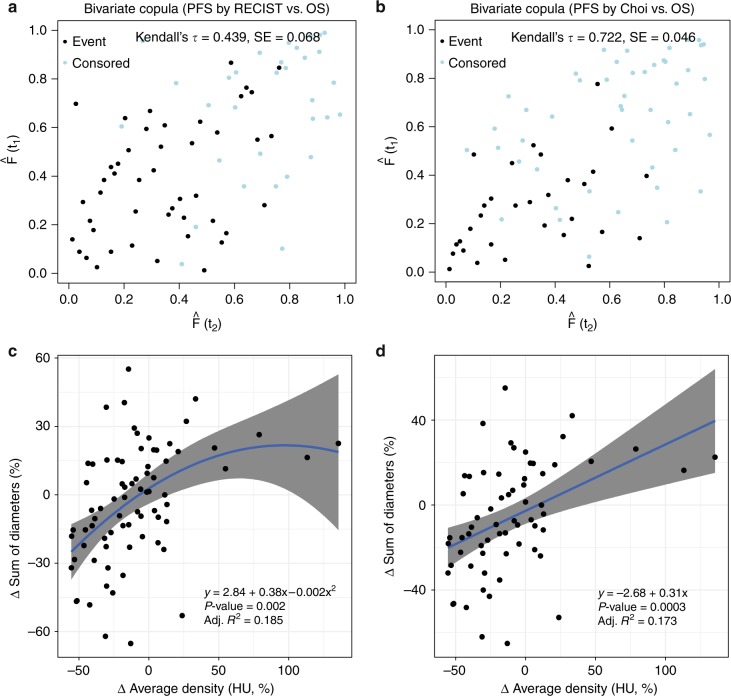
**a** Scatter plot of the bivariate survival copulas (PFS by RECIST vs OS); **b** scatter plot of the bivariate survival copulas (PFS by Choi vs OS). Interpretation of axes (**a**, **b**): progression-free survival (vertical) and overall survival (horizontal). **c** Δ Sum of diameters (%) and average Δ of tumour density (%) at 6 months; **d** Δ sum of diameters (%) and average Δ of tumour density (%) at 3 months. HU Hounsfield units, PFS progression-free survival, OS overall survival, SE standard error

During the follow-up period, 73 progression events were detected by RECIST and 64 by Choi criteria. The median PFS centrally evaluated were 11.42 months (95% confidence interval [CI], 9.7–15.9) as defined by RECIST and 15.8 (95% CI, 13.9–25.7) as per Choi criteria. PFS by Choi exhibited greater correlation with OS (Kendall’s τ = 0.722, standard error [SE] = 0.046) than PFS by RECIST (Kendall’s τ = 0.439, SE = 0.068) (see scatter plot of bivariate survival copulas in Fig. 1a, b). Of note, investigator-evaluated PFS was 14.5 months (95% CI, 11.8–18.0).

### Evaluation of tumour response in the first six months

**Fig. 2:**
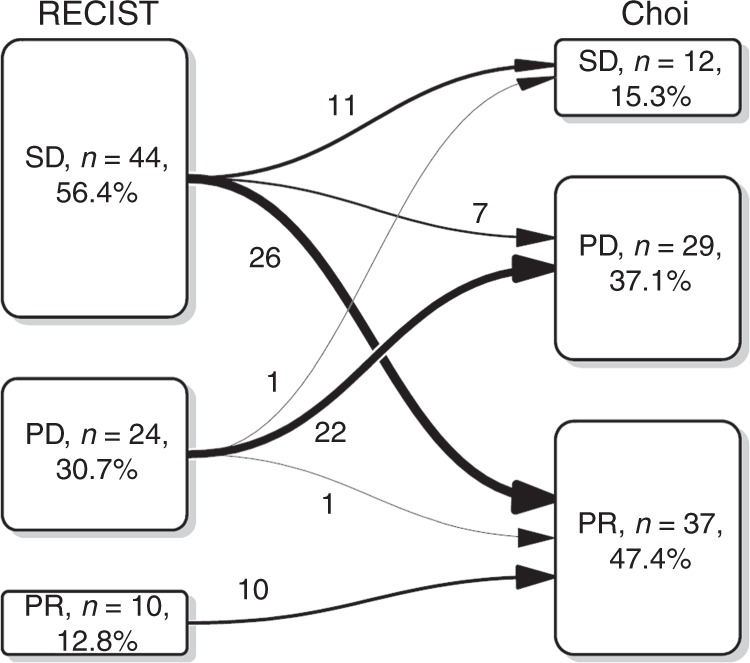
Transition plot with the reclassification between RECISTv1.1 and Choi criteria. n number, PD progressive disease, PR partial response, SD stable disease

Of the 85 subjects with a valid baseline CT, 78 had a CT at 6 months. At this time point, agreement between RECIST and Choi was weak (Cohen’s Kappa for 2 Raters, 0.392, *p* < 0.001). The transition diagram for both methods is shown in Fig. 2. According to RECIST, 12.8% attained a partial response (PR), 56.4% stable disease (SD) and 30.7% progressive disease (PD). When Choi criteria were applied, the main change was the increase in the percentage of PR, up to 47.4%, at the expense of decreasing the rate of SD to 15.3%, and of a slight increment in the percentage of PD (37.1%).

**Fig. 3:**
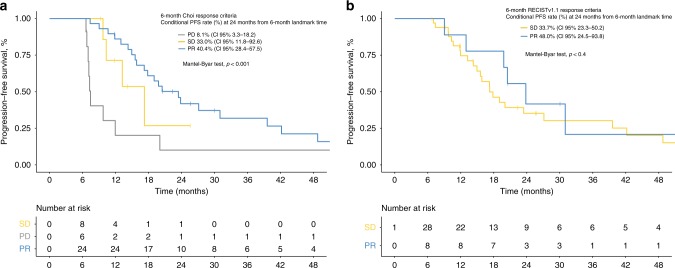
**a** Kaplan–Meier curves for conditional progression-free survival from 6-month landmark time stratified by Choi. **b** Kaplan–Meier curves for conditional progression-free survival from 6-month landmark time by RECIST. It does not include the PD category by RECIST as it coincides with the endpoint. CI confidence interval, PD progressive disease, PFS progression-free survival, PR partial response, SD stable disease

Supplementary Table 2 summarises the association of the 6-month tumour response assessment with survival endpoints. Landmark survival curve estimates are reported in Fig. 3. Choi criteria are seen to be significantly effective at predicting both PFS and OS (Mantel–Byar tests, *p* ≤ 0.001, in both cases). Evaluation by RECIST was associated significantly with OS. On the other hand, no statistical evidence was found to favour the association between tumour response per RECIST and PFS (Mantel–Byar test, *p* = 0.4). It must be remembered that, by definition, this analysis only contemplated two categories (PR and SD), as the RECIST-based PD endpoint coincided with the date of the CT in remaining cases that were found to be in progression (infinite, non-calculable hazard ratio [HR]). In contrast, the Choi-based PD (*n* = 29) identified a group at greater risk for progression as per RECIST (HR 2.55, 95% CI, 1.09–5.96) and for death (HR 2.44, 95% CI, 0.99–5.97). Tumour progression, by both RECIST and Choi criteria, increased the risk of death with an equivalent magnitude of effect in both cases. However, had only morphological criteria been applied, 7/29 patients with PD by Choi (24%) would have been classified as SD by RECIST. This would have overestimated these subjects’ prognosis, given that the 24-month OS rate (landmark) was 81.7% (95% CI, 69.6–91.1%) for SD as per RECIST, versus 50.1% (95% CI, 33.7–67.5%) for PD according to Choi criteria (see Supplementary Table 2). Furthermore, the median PFS in these seven subjects was 13.4 months when the endpoint was defined by RECIST versus 5.5 months as per Choi, which potentially entails 7.9 months of missed opportunity to change treatment

Likewise, 26/44 (59%) with RECIST SD were reclassified as PR by Choi. Applying only RECIST led to a discreet underestimation of prognosis, since the 24-month OS rate (landmark) was 91.8% (95% CI, 83.3–100%) for Choi PR, somewhat higher than RECIST-calculated SD. Nevertheless, the increase in the PR rate after applying Choi criteria did not devalue the prognostic meaning of response, which continued to exert an equivalent favourable effect, as per both Choi and RECIST. Thus, the 24-month PFS landmark for patients with Choi and RECIST PR was 40.4% (95% CI, 28.4–57.5%) and 48.0% (95% CI, 24.5–93.8%), respectively. Individuals with PR according to Choi had better PFS and OS, with HR 0.69 (95% CI, 0.29–1.61) and 0.86 (95% CI, 0.34–2.16), respectively, versus those with SD, but the results were not statistically significant.

### Evaluation of tumour response in the first 3 months

Subsequently, the ‘earlier’ evaluation performed in 63 patients after a median of 2.8 months (range, 1.1–4.4) was predictive of PFS/OS (see Supplementary Table 3). In this scenario, PD by RECIST was observed to have a more significant and greater effect on the risk of death (HR for OS of 2.22, 95% CI, 1.13–4.25) compared to PD defined by Choi criteria (HR for OS of 1.28, 95% CI, 0.58–2.83). However, no statistical evidence was found that pointed toward early evaluation by RECIST significantly predicted PFS (Mantel–Byar test, *p* = 0.3), although we must bear in mind that, by definition, this analysis only has two RECIST-defined categories (PR and SD; the category PD by RECIST coincides with the endpoint). Choi criteria evaluated at 3 months displayed a significant association with PFS (Mantel–Byar test, *p* = 0.04), but not OS (Supplementary Table 3).

### Evaluation of tumour growth rate and attenuation variation rate

Finally, the relation between tumour size and AVR was examined, as was the influence of these parameters on prognosis. First of all, scatter plots were constructed that yield a graphic illustration of the correlation between relative variations in HU and DVR (Δ sum of diameters, %), at 6 and 3 months (Fig. 1c, d, respectively). These scatter plots reveal that changes in attenuation are significantly associated with variations in size, accounting for approximately 18% of their variation. In both cases, the slopes are moderate, as these are slow-growing tumours. As expected, the DVR exhibited a very high correlation with the TGR (Kendall’s τ = 0.960). Likewise, the AVR explains 21% of the variability in tumour volume at 6 months.

**Fig. 4:**
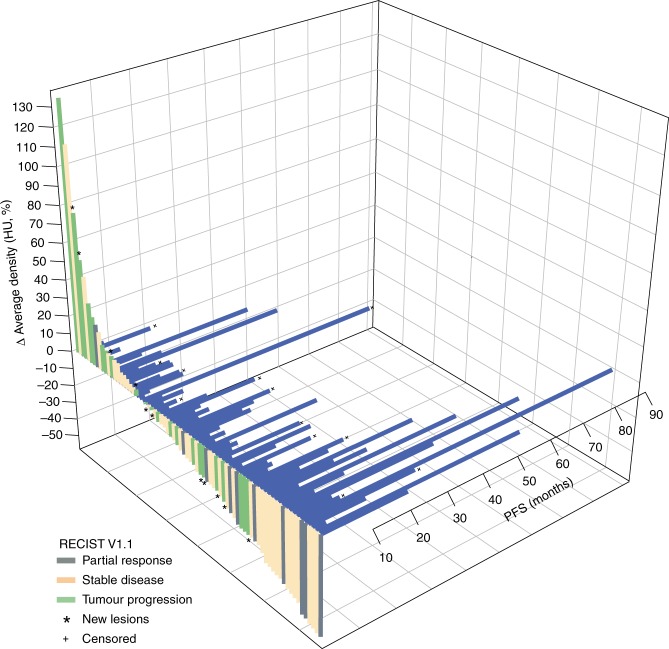
3D-waterfall plot with the Δ in the average of HU, RECIST v1.1 and PFS. HU hounsfield units, PFS progression-free survival

To demonstrate how these changes projected on evaluations of response, spider plots were drawn that illustrate the cross relationship between: (1) DVR versus Choi criteria, and (2) AVR versus RECIST (Supplementary Fig. 1A, B, respectively). In this case, given the slow growth rate, no significant changes in size were observed between time point 0 and 3 or 6 months (Wilcoxon test, *p* > 0.1). In contrast, attenuation did decrease significantly in both spans (Wilcoxon test, *p* < 0.001). Figure 4 is a 3D-waterfall plot that illustrates the relation between RECIST, AVR, and PFS in each PanNET.

Next, we assessed whether changes in DVR, TGR and AVR could predict prognosis during treatment with sunitinib. Supplementary Table 4 shows the association between DVR, TGR and AVR with survival endpoints at 3 and 6 months. While both parameters are associated with PFS and OS in univariate analyses, only TGR is an independent prognostic factor in the multivariate model for both endpoints (see Supplementary Table 4).

## Discussion

Proper monitoring of response to antineoplastic treatments is fundamental to reduce toxicity and costs. In the specific case of sunitinib, this antiangiogenic drug induces biological changes in the tumours, that are visible on CTs, such as variations in density,^10,18^ and produces a reduction in tumour size in most cases, although these regressions rarely reach the threshold of PR by RECIST.^6^ Choi criteria are based on subtle changes in attenuation or size, theoretically making them a more adequate method by which to track sunitinib’s effect in vivo (Smith et al., 2010; Faivre et al., 2012; Raymond et al., 2018). Nonetheless, there is scant experience with monitoring treatment effect using Choi criteria in PanNETs treated with antiangiogenics.^10,16,18^

In other hypervascular tumours, such as hepatocellular carcinoma, kidney cancer or gastrointestinal stromal tumours, Choi criteria have been used to assess response to tyrosine-kinase inhibitors having an antiangiogenic effect, such as sorafenib, axitinib, sunitinib, pazopanib, etc.^31^ Other criteria have also been developed to respond to the complexities specific to each disease^32,33^ and treatment modality, such as the modified RECIST in hepatocellular carcinoma treated with chemoembolisation or MASS (morphology, attenuation, size and structure) criteria in renal cancer treated with antiangiogenics.^34,35^

In the CRIPNET_GETNE1504 study, we have observed that assessment of response via RECIST v1.1 at 6 months categorises more than half of the series as SD, with relatively few PR. In our cohort, tumours with SD as per RECIST comprised a heterogeneous group from a prognostic perspective. This was presumably due to the fact that tumour growth was typically slow, regardless of the efficacy of sunitinib. When tumours were appraised by Choi criteria, the most noteworthy consequence was the dramatic increase in PR rate (Δ + 34.6%). All told, more than half of the cases of RECIST SD were reclassified as PR by Choi. The possibility of re-assessing sunitinib’s activity in PanNETs is consistent with earlier information in the literature.^10,16,18^ Our data point toward response as defined by Choi criteria, despite it being more ‘permissive’, had a favourable effect on PFS/OS comparable to RECIST-determined response. Since no evidence was found to endorse a substantial decline in prognostic value, the conclusion is that Choi was able to capture a clinically significant biological effect that went unnoticed by RECIST. Therefore, most individuals in PR as per Choi criteria in our series, in all likelihood actually benefitted from the drug.

Although the percentage of subjects with RECIST SD reclassified as PD was relatively low, had Choi criteria not been applied, prognosis would have been overestimated in some 16%. However, the impact of PD according to Choi criteria is complex and subtle. On the one hand, Choi criteria employ a more restrictive threshold of PD (10% versus 20% with RECIST). However, the definition crucially excludes those tumours that, despite having grown, meet the criterion of PR based on decreased attenuation. The overall result was that, paradoxically, despite the slight increase in PD at 6 months with Choi criteria, PFS evaluated by these same criteria was higher than RECIST PFS. The reason for this was that, while there were more initial progressions with Choi criteria, the effect was offset by tumours that maintained hypo-attenuation at 12 months, despite their slow growth. This latter observation is in line with the data from the phase IV sunitinib trial.^16^ The reduction of the cut-off (from 20 to 10%) improved the OS prediction overall, but did not affect the specificity of the definition of PD. Lamarca et al. recently published similar data.^15^

Therefore, the crux of the matter is what definition of PFS is the most valid for use as a surrogate for OS in this setting. Imaoka et al. previously considered that RECIST-defined PFS was an acceptable OS surrogate in PanNETs.^36^ In contrast, our data reveal that the correlation between PFS according to Choi and OS is intermediate, and low, in the case of RECIST-based PFS. In line with pre-set criteria, this is compatible with a lack of validity of the latter surrogate.^37^ Nevertheless, correlation between surrogates and patient-relevant endpoints alone should not be the sole criterion to be contemplated in the case of indolent tumours, such as neuroendocrine tumours, with several effective treatment options after first-line. Be that as it may, Choi PFS displayed a slightly better correlation with OS, although further studies are needed to prove this surrogate’s validity.

Quite a different issue is whether the dichotomisation of response entails loss of information, by ignoring gradualness.^17^ AVR and TGR correctly predicted RECIST-based PFS. Said effect was already present in studies at 3 months; hence, AVR and TGR can be early markers of clinically significant activity of the drug. These measurements of variation by unit of time worked better than density- or size-related variations. Furthermore, both were non-linearly interrelated; consequently, in multivariate analyses, only TGR predicted survival endpoints. However, several nuances may limit the practical use of TGR in some patients. First of all, the difficulty in discerning the prognostic significance of slight differences in size must be taken into account, especially since they are the ones that occur in most patients. In addition, it cannot be ruled out that AVR might modulate the effect of discrete growth, insofar as hypo-attenuation translates into sustained antiangiogenic activity. Nonetheless, the reader must be aware that this study is not statistically powered to detect this kind of subgroup effect. It must also be remembered that in patients not treated with antiangiogenics, hypoattenuated PanNETs are generally associated with tumour de-differentiation and poor prognosis.^5^

The CRIPNET_GETNE1504 study has certain limitations. Given the low incidence of PanNETs, the sample size is relatively small. Despite this, it is one of the largest series reported to date.^10,16,18^ Secondly, the decision as to whether discontinue sunitinib or not was based on each centre’s clinical practice, separate from the centralised radiological criterion, which may have influenced the estimation of PFS. The reader must likewise be aware that, while certain minimum quality criteria were required, and 22 cases were excluded on the basis of insufficient imaging quality, the studies were conducted in accordance with the local protocols of each centre. Choi criteria should be generalised by means of the rigorous application of standardised protocols. This calls for prior consensus across the radiodiagnostic and other departments involved in the management of patients with neuroendocrine tumours. Finally, interobserver agreement between the two radiologists who analysed response was not examined. Nonetheless, earlier studies point toward the level of concordance is generally high.^38,39^

In conclusion, in advanced PanNETs treated with sunitinib, RECIST as the sole method of monitoring response entails an imprecise or erroneous prognostic prediction in almost half of the patients. RECIST PFS was a worse surrogate for OS than Choi-estimated PFS. In contrast, Choi criteria capture the activity of sunitinib in a clinically significant manner and suit the follow-up of slow-growing PanNETs. If the data from this study are confirmed through data from clinical trials, Choi criteria should be considered as standard for monitoring advanced PanNETs response to antiangiogenic therapy.

## Supplementary information

Supplemental Material

## Data Availability

All the data generated or analysed in this study are included in the manuscript or the supplementary information.
