# Supplementary material for: Evaluating radiological response in pancreatic neuroendocrine tumours treated with sunitinib: comparison of Choi versus RECIST criteria (CRIPNET_ GETNE1504 study)
Source: Br J Cancer. 2019 Sep 3;121(7):537–44. doi: 10.1038/s41416-019-0558-7 (PMC6889276; doi:10.1038/s41416-019-0558-7)

Supplementary Table 1. Definitions of RECIST/Choi criteria used in the analyses of the images of computed tomography

CRITERIA	RECIST 1.1	CHOI
**CR**	Disappearance of all target lesions. Any pathological lymph nodes (whether target or non-target) must have reduction in short axis to <10 mm	Disappearance of all target lesion(s)
**PR**	Decrease ≥30% in the sum of the diameters of target lesions taking as reference the baseline sum. No new lesion(s)	Decrease in tumor size ≥10% or decrease in tumor density ≥15% on CT
**SD**	Neither response nor progression. No new lesion(s)	Does not meet the criteria for CR, PR or PD
**PD**	Increase ≥20% in the sum of the diameters of target lesions taking as reference the smallest sum measured during follow-up (nadir) and ≥5mm in absolute value and/or appearance of new unequivocally metastatic lesion(s)	Increase ≥10% in tumor size and does not meet PR criteria by tumor density New lesions

Abbreviations: RECIST= Response Evaluation Criteria In Solid Tumors, CR= complete response, PR= partial response, SD= stable disease, PD= progressive disease.

Supplementary Table 2. Summary of effects of 6-month tumor response assessment on PFS/OS times

	Choi criteria	RECIST v1.1
12-month landmark PFS (CI 95%)		
PR	73.1 (58.6-85.8)	90.0 (73.2-100)
SD	61.9 (38.2-99.9)	65.8 (53.5-80.4)
PD	15.3 (6.6-29.1)	-
24-month landmark PFS (CI 95%)		
PR	40.4 (28.4-57.5)	48.0 (24.5-93.8)
SD	33.0 (11.8-92.6)	33.7 (23.3-50.2)
PD	8.1 (3.3-18.2)	-
12-month landmark OS (CI 95%)		
PR	100 (NA)	100 (NA)
SD	100 (NA)	94.8 (85.9-99.3)
PD	66.3 (51.3-81.7)	67.2 (50.4-83.4)
24-month landmark OS (CI 95%)		
PR	91.8 (83.3-100.0)	100 (NA)
SD	75.0 (54.1-100.0)	81.7 (69.6-91.1)
PD	50.1 (33.7-67.5)	48.1 (32.2-66.1)
Median PFS from the response assessment (months, CI 95%) ‡		
PR	14.2 (10.7-33.4)	18.1 (14.22-NA)
SD	9.7 (5.8-NA)	10.1 (8.2-21.7)
PD	1.0 (1.0-1.0)	-
Hazard ratio for PFS (CI 95%)		
PR	0.69 (0.29-1.61)	0.71 (0.31-1.66)
SD	Ref.	Ref.
PD	2.55 (1.09-5.96)	-
Hazard ratio for OS (CI 95%)		
PR	0.86 (0.34-2.16)	0.80 (0.30-2.11)
SD	Ref.	Ref.
PD	2.44 (0.99-5.97)	2.64 (1.46-4.78)

Abbreviations: PFS= progression-free survival, RECIST= Response Evaluation Criteria In Solid Tumors; CI= confidence interval, PR= partial response, SD= stable disease, PD=progressive disease, Ref.= reference category, NA= not available. ‡ The median PFS from time zero is calculated by adding the time from start to the time of CTs for response assessment.

Supplementary Table 3. Cox PH regression for PFS and OS as per RECIST/Choi at 3 months (univariate analysis)

**End point, PFS**	HR (CI 95%)	P-value
Choi		
PR	0.86 (0.39-1.89)	0.717
SD	Ref.	-
PD	2.89 (0.95-8.81)	0.060
RECIST v1.1		
PR	0.61 (0.25-1.46)	0.273
SD	Ref.	-
PD	-	-
**End point, OS**	HR (CI 95%)	P-value
Choi		
PR	0.63 (0.31-1.31)	0.223
SD	Ref.	-
PD	1.28 (0.58-2.83)	0.529
RECIST v1.1		
PR	1.26 (0.51-3.11)	0.611
SD	Ref.	-
PD	2.22 (1.13-4.25)	0.020
Abbreviations: Ref.= reference category, PR= partial response, SD= stable disease, PD= progressive disease, HR= hazard ratio.		

Supplementary Table 4. Cox PH regression for PFS and OS evaluating the effect of TGR and AVR quantified at 3 and 6 months

3-month assessment				
	**Univariate**		**Multivariate**	
	**End point, PFS by RECIST**			
	HR (CI 95%)	*P-value*	HR (CI 95%)	*P-value*
DVR	0.98 (0.96-0.99)	0.011	-	-
TGR	0.99 (0.98-0.99)	0.005	0.99 (0.98-0.99)	0.022
AVR	0.98 (0.98-0.99)	0.030	0.99 (0.98-1.00)	0.259
	**End point, OS**			
	HR (CI 95%)	*P-value*	HR (CI 95%)	P-value
DVR	0.98 (0.98-1.01)	0.865	-	-
TGR	0.99 (0.99-1.01)	0.917	0.99 (0.98-1.01)	0.820
AVR	0.99 (0.98-1.01)	0.263	1.00 (0.99-1.00)	0.255
6-month assessment				
	**End point, PFS by RECIST**			
	HR (CI 95%)	*P-value*	HR (CI 95%)	*P-value*
DVR	0.87 (0.82-0.93)	<0.001	-	-
TGR	0.95 (0.93-0.97)	<0.001	0.96 (0.94-0.98)	0.001
AVR	0.95 (0.92-0.98)	0.001	0.97 (0.94-1.01)	0.204
	**End point, OS**			
	HR (CI 95%)	*P-value*	HR (CI 95%)	*P-value*
DVR	0.93 (0.87-0.98)	0.014	-	-
TGR	0.97 (0.95-0.99)	0.007	0.97 (0.95-0.99)	0.043
AVR	0.96 (0.92-1.00)	0.079	0.98 (0.94-1.02)	0.380

Abbreviations: HR= hazard ratio, AVR= attenuation variation rate, DVR= diameter variation rate, TGR= tumor growth rate, PFS= progression-free survival, OS= overall survival, CI= confidence interval.

Supplementary Table 5. Institutional ethics committees that have approved this study

1 Hospital Universitario Central de Asturias

2 Hospital Universitario Morales Meseguer de Murcia

3 Hospital Universitario 12 de Octubre de Madrid

4 Hospital Universitario La Paz de Madrid

5 Hospital Universitario Ramón y Cajal de Madrid

6 Hospital Universitario Virgen del Rocío de Sevilla

7 Hospital Universitario Vall d'Hebron de Barcelona

8 Hospital Universitario Miguel Servet de Zaragoza

9 Hospital Universitario Marqués de Valdecilla de Santander

10 Complejo Hospitalario de Navarra de Pamplona

Supplementary Figure 1. Spider plots with size and attenuation variation. Abbreviations: mm= millimeters, HU= Hounsfield units. 4A: size variation by Choi criteria, 4B: attenuation variation by RECIST

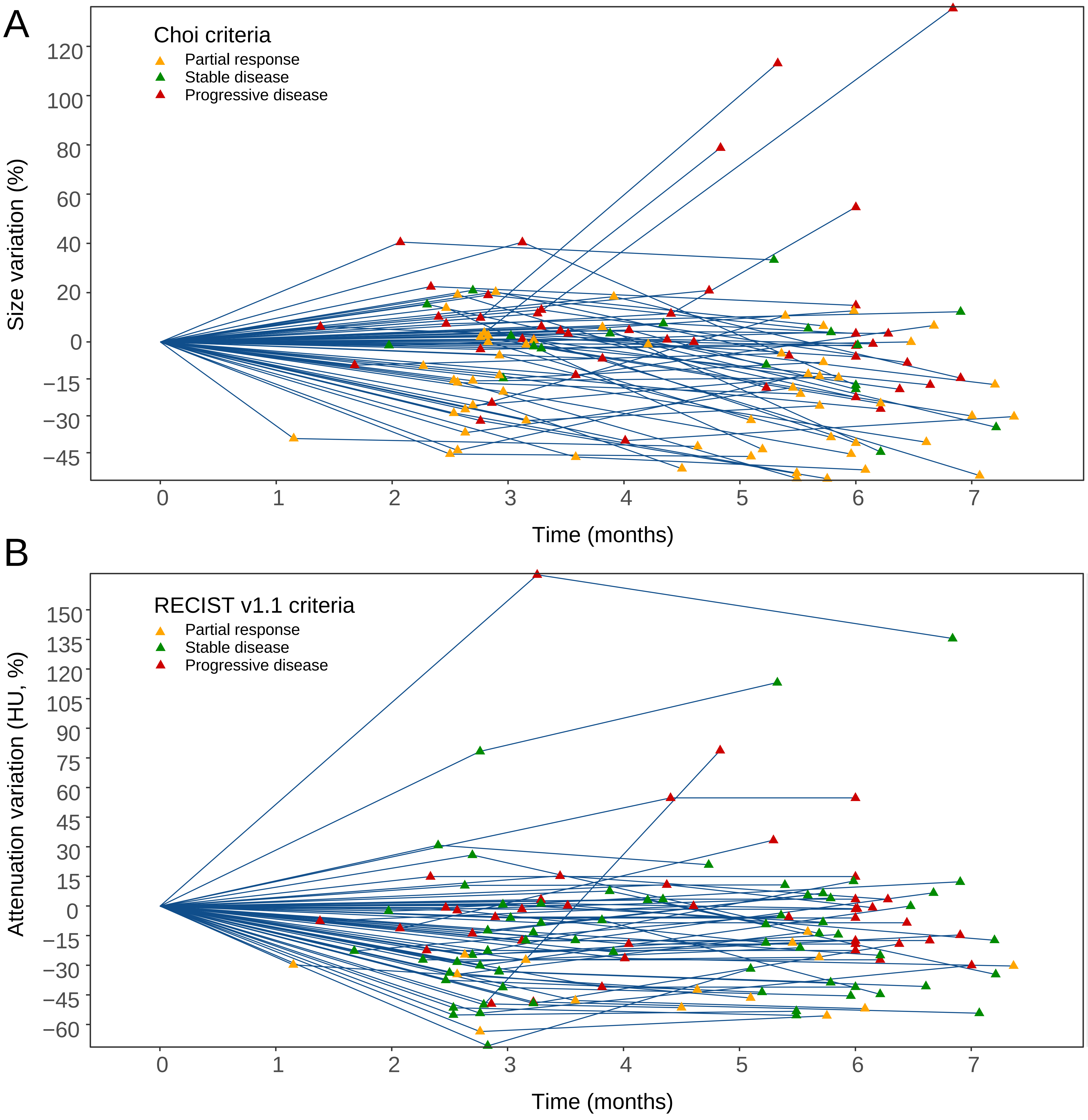

Supplement: Supplementary file 1 — Supplemental Material [file 41416_2019_558_MOESM1_ESM.pdf]
